# Negative and positive experiences of caregiving among family caregivers of older blunt trauma patients

**DOI:** 10.1371/journal.pone.0275169

**Published:** 2022-10-10

**Authors:** Ting-Hway Wong, Timothy Xin Zhong Tan, Lynette Ma Loo, Wei Chong Chua, Philip Tsau Choong Iau, Arron Seng Hock Ang, Jerry Tiong Thye Goo, Kim Chai Chan, Hai V. Nguyen, Nivedita V. Nadkarni, David Bruce Matchar, Dennis Chuen Chai Seow, Yee Sien Ng, Angelique Chan, Stephanie Fook-Chong, Tjun Yip Tang, Marcus Eng Hock Ong, Rahul Malhotra

**Affiliations:** 1 Health Services and Systems Research, Duke-NUS Graduate Medical School, Singapore, Singapore; 2 Department of General Surgery, Singapore General Hospital, Singapore, Singapore; 3 Department of Emergency Medicine, Singapore General Hospital, Singapore, Singapore; 4 Department of General Surgery, National University Hospital, Singapore, Singapore; 5 Trauma Service, Tan Tock Seng Hospital, Singapore, Singapore; 6 Accident & Emergency, Changi General Hospital, Singapore, Singapore; 7 Department of General Surgery, Khoo Teck Puat Hospital, Singapore, Singapore; 8 Emergency Medicine Department, Ng Teng Fong General Hospital, Singapore, Singapore; 9 School of Pharmacy, Memorial University of Newfoundland, St. John’s, Canada; 10 Centre for Quantitative Medicine, Duke-NUS Graduate Medical School, Singapore, Singapore; 11 Department of Geriatric Medicine, Singapore General Hospital, Singapore, Singapore; 12 Department of Rehabilitation Medicine, Singapore General Hospital, Singapore, Singapore; 13 Centre for Ageing Research and Education, Duke-NUS Graduate Medical School, Singapore, Singapore; 14 Department of Vascular Surgery, Singapore General Hospital, Singapore, Singapore; Chung Shan Medical University, TAIWAN

## Abstract

**Objectives:**

Family caregivers play a fundamental role in the care of the older blunt trauma patient. We aim to identify risk factors for negative and positive experiences of caregiving among family caregivers.

**Design:**

Prospective, nationwide, multi-center cohort study.

**Setting and participants:**

110 family caregivers of Singaporeans aged≥55 admitted for unintentional blunt trauma with an Injury Severity Score (ISS) or New Injury Severity Score (NISS)≥10 were assessed for caregiving-related negative (disturbed schedule and poor health, lack of family support, lack of finances) and positive (esteem) experiences using the modified-Caregiver Reaction Assessment (m-CRA) three months post-injury.

**Methods:**

The association between caregiver and patient factors, and the four m-CRA domains were evaluated via linear regression.

**Results:**

Caregivers of retired patients and caregivers of functionally dependent patients (post-injury Barthel score <80) reported a worse experience in terms of disturbed schedule and poor health (β-coefficient 0.42 [95% Confidence Interval 0.10, 0.75], p = .01; 0.77 [0.33, 1.21], p = .001), while male caregivers and caregivers who had more people in the household reported a better experience (-0.39 [-0.73, -0.06], p = .02; -0.16 [-0.25, -0.07], p = .001). Caregivers of male patients, retired patients, and patients living in lower socioeconomic housing were more likely to experience lack of family support (0.28, [0.03, -0.53], p = .03; 0.26, [0.01, 0.52], p = .05; 0.34, [0.05, -0.66], p = .02). In the context of lack of finances, caregivers of male patients and caregivers of functionally dependent patients reported higher financial strain (0.74 [0.31, 1.17], p = .001; 0.84 [0.26, 1.43], p = .01). Finally, caregivers of male patients reported higher caregiver esteem (0.36 [0.15, 0.57], p = .001).

**Conclusions and implications:**

Negative and positive experiences of caregiving among caregivers of older blunt trauma patients are associated with pre-injury disability and certain patient and caregiver demographics. These factors should be considered when planning the post-discharge support of older blunt trauma patients.

## Introduction

Family caregiving, linked with both negative and positive experiences for the caregiver, is an important aspect of the multidisciplinary care for older persons [1–4]. Older blunt trauma patients often present with a unique combination of emergency conditions on a background of physical frailty and cognitive impairment, and are susceptible to higher adjusted morbidity, mortality and readmission [5–8]. This has been attributed to decreased physiologic reserve and increased vulnerability to external stressors [6, 7, 9].

Previous results from our study of older blunt trauma patients showed that pre-injury baseline frailty was associated with post-discharge functional decline and increased health services utilization [9, 10]. Studies on caregivers of patients with dementia have highlighted caregiver burden as a major contributor to post-discharge healthcare utilization [11]. Identifying caregiver and patient factors which predispose caregivers of older blunt trauma patients to negative experiences of caregiving is therefore important and could help guide the planning and prioritization of future interventions to relieve caregiver stress [12–14].

Therefore, we sought to identify factors influencing the caregiving experience among family caregivers of older blunt trauma patients. We hypothesized that the caregiving experience for such patients would be affected by caregiver demographics, household characteristics and patient demographics, in addition to patient injury severity, injury pattern, and pre-injury comorbidities and function.

## Materials and methods

### Study population and data collection

Singapore is a rapidly aging Asian nation with a life expectancy at birth of 83.1 years and a population of 5.5 million, of whom 24.6% are 55 years and older, compared to 17.3% worldwide [15, 16].

In this prospective, nationwide, multi-center cohort study of Singaporean residents (citizens or permanent residents), patients admitted via all public hospital emergency departments were screened via the Singapore National Trauma Registry (NTR). Injury Severity Score (ISS) [17], New Injury Severity Score (NISS) [18], Abbreviated Injury Scale (AIS) [19] and Revised Trauma Score (RTS) [20] data were retrieved from NTR offices at the respective study sites. Demographics (age, gender, race, housing type and employment status) were also drawn from the NTR and verified in the questionnaire.

Primary caregivers (self-identified during recruitment as having primary responsibility for care) of Singapore residents aged ≥55 years admitted for ≥48 hours after unintentional blunt trauma (cases of assault and self-harm were excluded) from Mar 2016 to Jul 2018 with an Injury Severity Score (ISS) or New Injury Severity Score (NISS) ≥10 and survived index hospitalization, were invited to participate in the study. Recruitment of patients was carried out during index admission with written consent. Consent was obtained from patients who were deemed by the research team to meet mental capacity: (1) able to understand information provided regarding the decision, (2) retain the information, (3) appreciate and analyze this information in order to come to a conclusion, and (4) communicate aforementioned conclusion through any means. For patients who did not have mental capacity to consent, or who were unable to respond appropriately to questionnaires, their caregivers were approached for the caregiver questionnaire arm of the study. Patients and caregivers were not approached if: the primary attending physician did not agree to the study team approaching the patient or caregiver, if the patient was not expected to survive the admission, or if the patient could not give consent and there was no caregiver. The first author’s Institutional Review Board granted ethical approval for the study (SingHealth IRB Reference 2015/2590).

Primary family caregivers were evaluated via in-person survey for caregiving related negative (disturbed schedule and poor health; lack of family support; lack of finances) and positive (esteem) experiences using the 21-item modified-Caregiver Reaction Assessment (m-CRA) three months post-injury [21, 22].

### Statistical analysis

The association between caregiver and patient factors, and the four m-CRA domain scores (range: 1–5) were evaluated via linear regression. A higher domain score indicates a worse status for negative domains and a better status for the positive domain. For each domain, factors with significant associations (p < .05) on univariate regression were included in a multivariable linear regression model. Stata 15.1 was used.

## Results

Of the 128 caregivers who agreed to participate in the study, 110 caregivers (85.9%) completed the m-CRA at three months post-injury and were included for analysis. Most caregivers were female (70, 63.6%) with a median age of 55 (IQR 47–65) and an ethnicity distribution representative of the general population of Singapore (**Table 1**). Caregivers were mostly spouses (38, 34.5%) or children (son 30, 27.3%; daughter 30, 27.3%) of the patient. More than half the patients had no formal education (20, 15.6%) or did not complete primary school (54, 42.2%).

**Table 1:** Characteristics of older blunt trauma patients and their caregivers (n = 110).

**Demographics (Caregivers)**		**Number (%) or Median (IQR)**
Age	Years, Median (IQR)	55 (47–65)
Gender	Male	40 (36.4)
	Female	70 (63.6)
Ethnicity	Chinese	89 (80.9)
	Malay	14 (12.7)
	Indian	6 (5.5)
	Others	1 (0.9)
Relationship with care recipient	Spouse	38 (34.5)
	Son	30 (27.3)
	Daughter	30 (27.3)
	Sibling	3 (2.7)
	Others*	10 (9.1)
Living with care recipient	Yes	97 (88.2)
Modified-Caregiver Reaction Assessment score, Median (IQR)	Disturbed Schedule and Poor Health	2.63 (2.13–3.50)
	Lack of Family Support	2.00 (1.80–2.40)
	Lack of Finances	2.25 (2.00–3.50)
	Caregiver Esteem	1.17 (0.67–1.50)
**Demographics (Patients)**		**Number (%) or Median (IQR)**
Age	Years, Median (IQR)	77 (66–86)
Gender	Male	61 (55.5)
	Female	49 (44.6)
Ethnicity	Chinese	90 (81.8)
	Malay	14 (12.7)
	Indian	6 (5.5)
Housing Type	1–2 room public	9 (8.2)
	3-room public	19 (17.3)
	4-room public	41 (37.3)
	≥5-room public	24 (21.8)
	Private	17 (15.5)
Employment status	Full-time	23 (20.9)
	Part-time	4 (3.6)
	Homemaker	21 (19.1)
	Retired	62 (56.4)
Highest level of education	Did not complete Primary / None	16 (14.6)
	Primary	47 (42.7)
	Secondary	37 (33.6)
	Tertiary	10 (9.1)
Living arrangement	Living with someone	105 (95.5)
	Living alone	5 (4.5)
	Number of people in household	3 (1–7)
	Foreign domestic worker at home	31 (28.2)
Interviewee	Patient	57 (51.8)
	Caregiver (Patient unable to answer)	53 (48.2)
Injury Severity Score (ISS)	10–15	67 (60.9)
	16–24	27 (24.6)
	≥25	16 (14.5)
ISS regions with Abbreviated Injury Scale (AIS) of ≥3	Head and neck	65 (59.1)
	Face	0
	Thorax	21 (19.1)
	Abdomen/Pelvis	8 (7.3)
	Extremities	25 (22.7)
	External	0
Anatomical polytrauma	AIS≥3 in ≥2 ISS regions	12 (10.9)
Revised Trauma Score (RTS)	<7.841 (abnormal)	6 (5.5)
	7.841 (normal)	104 (94.5)
Mechanism of injury	Low fall < = 0.5m	80 (72.7)
	High fall >0.5m	6 (5.5)
	Motor Vehicle Injury	24 (21.8)
Frail	Modified Fried’s Criteria≥3	25 (22.7)
Functional dependence	Barthel Score <80 (dependent)	18 (16.4)
Mini Mental State Examination (MMSE)	MMSE≤19 (or unable)	69 (62.7)
	MMSE>19	41 (37.3)
Charlson Comorbidity Index (CCI)	0	33 (30.0)
	1	25 (22.7)
	2	15 (13.6)
	≥3	37 (33.6)

*4 daughters-in-law, 2 grandchildren, 2 aunts, 1 son-in-law, 1 godson

Just over one-third the patients (43, 39.1%) were severely or critically injured (ISS ≥16), with the remainder moderately injured (ISS 10–15). The proportion of anatomical polytrauma patients (AIS≥3 for 2 or more ISS regions) was 10.9% (12 patients). In terms of pattern of injury, the three most common regions with significant injury (defined as AIS score ≥3) were the head (65, 59.1%), extremities (25, 22.7%), and thorax (21, 19.1%).

Low fall patients (defined as ≤0.5m) constituted the majority of patients (93, 72.7%), followed by patients of motor vehicle accidents (28, 21.9%), and higher-level fallers (7, 5.5%). Twelve patients (21.9%) were determined to be frail as per modified Fried’s criteria.

### Disturbed schedule and poor health

Caregivers of retired patients (versus working), and caregivers of functionally dependent patients (Barthel’s score <80 post-injury) reported a worse experience (β-coefficient: 0.42, 95% Confidence Interval [CI] 0.10–0.75, p = .01; β-coefficient 0.77, 95% CI 0.33–1.21, p = .001) (**Table 2**). Male (versus female) caregivers reported a better experience, as did caregivers with more people in the household (β-coefficient -0.39, 95% CI -0.73- -0.06, p = .02; β-coefficient: -0.16, 95% CI -0.25- -0.07, p = .001).

**Table 2:** Factors associated with the domains of the modified-caregiver reaction assessment.

	Disturbed Schedule and Poor Health				Lack of Family Support				Lack of Finances				Caregiver Esteem^†^	
	Univariate		Multivariable		Univariate		Multivariable		Univariate		Multivariable		Univariate	
	Coeff (95% CI)	p	Coeff (95% CI)	p	Coeff (95% CI)	p	Coeff (95% CI)	p	Coeff (95% CI)	p	Coeff (95% CI)	p	Coeff (95% CI)	p
Patient Characteristics*														
Male gender	0.23 (-0.14–0.59)	.22			**0.31 (0.05–0.57)**	**.02**	**0.28 (0.03–0.53)**	**.03**	**0.64 (0.19–1.09)**	**.01**	**0.74 (0.31–1.17)**	**.001**	**0.36 (0.15–0.57)**	**.001**
Retired	**0.48 (0.13–0.84)**	**.01**	**0.42 (0.10–0.75)**	**.01**	**0.33 (0.07–0.59)**	**.02**	**0.26 (0.01–0.52)**	**.05**					0.12 (-0.10–0.34)	.27
Age	0.01 (0.00–0.03)	.17			0.00 (-0.01–0.01)	.58			0.00 (-0.02–0.02)	.88			0.00 (-0.01–0.01)	.69
Living in highly or moderately subsidized housing (relative to private or minimally-subsidized housing)	0.33 (-0.08–0.75)	.12			**0.36 (0.06–0.66)**	**.02**	**0.34 (0.05–0.63)**	**.02**	0.50 (-0.02–1.02)	.07			0.04 (-0.21–0.29)	.74
Number of people in household	**-0.15 (-0.25- -0.04)**	**.01**	**-0.16 (-0.25- -0.07)**	**.001**	-0.02 (-0.10–0.05)	.56			0.00 (-0.14–0.13)	.95			-0.02 (-0.08–0.05)	.61
Charlson Comorbidity Index (CCI) ≥2	0.32 (-0.04–0.68)	.08			0.24 (-0.02–0.51)	.07			0.47 (0.02–0.92)	.04	0.39 (-0.04–0.82)	.08	0.13 (-0.09–0.34)	.25
Functionally Dependent: Barthel’s <80	**0.67 (0.20–1.15)**	**.01**	**0.77 (0.33–1.21)**	**.001**	0.25 (-0.11–0.61)	.17			**0.79 (0.18–1.39)**	**.01**	**0.84 (0.26–1.43)**	**.01**	-0.28 (-0.57–0.02)	.06
Mini-Mental-State Examination score (MMSE)≤19	0.35 (-0.03–0.72)	.07			0.24 (-0.03–0.51)	.08			0.31 (-0.16–0.78)	.19			0.09 (-0.14–0.31)	.44
Frailty (Modified Fried’s Criteria≥3)	-0.14 (-0.57–0.30)	.54			0.16 (-0.16–0.47)	.32			0.48 (-0.07–1.02)	.08			-0.21 (-0.47–0.04)	.11
**Caregiver Characteristics**														
Male gender	**-0.39 (-0.76- -0.02)**	**.04**	**-0.39 (-0.73- -0.06)**	**.02**	-0.10 (-0.37–0.18)	.50			-0.09 (-0.57–0.39)	.71			-0.03 (-0.26–0.20)	.78
Age	0.01 (-0.01–0.02)	.36			0.00 (-0.01–0.01)	.41			0.00 (-0.02–0.01)	.73				
Spouse	0.18 (-0.21–0.56)	.37			0.24 (-0.03–0.52)	.08			0.15 (-0.34–0.63)	.54				

*(Factors highlighted in bold are associated with one or more caregiver domains in multivariable models at 0.05 level of significance)

^†^Multivariable model not shown because only one factor was significant on univariate.

### Lack of family support

Caregivers of male patients, retired patients, and patients living in lower socioeconomic/more subsidized housing (versus private/minimally subsidized housing) were more likely to experience lack of family support (β-coefficient 0.28, 95% CI 0.03–0.53, p = .03; β-coefficient 0.26, 95% CI 0.01–0.52, p = .05; β-coefficient 0.34, 95% CI 0.05–0.66, p = .02).

### Lack of finances

Caregivers of male patients and caregivers of functionally dependent patients reported higher financial strain (β-coefficient 0.74, 95% CI 0.31–1.17, p = .001; β-coefficient 0.84, 95% CI 0.26–1.43, p = .01). Higher Charlson co-morbidity index was also associated with higher financial strain on univariate analysis (β-coefficient 0.47, 95% CI 0.02–0.92, p = .04), but not in the multivariable model.

### Esteem

Caregivers of male patients also reported higher caregiver esteem (β-coefficient 0.36, 95% CI 0.15–0.57, p < .01).

The patient’s educational level, injury severity, pattern of injury, mechanism of injury, frailty, and cognitive function were not associated with the caregiving experience in any domain.

## Discussion

Understanding the risk factors for negative and positive experiences of caregiving are important in the multidisciplinary care of older patients after blunt trauma, and in planning post-discharge support for caregivers. Good family function, social support, behavioral intervention and resilience skills are associated with reduced caregiver burden after injury [12–14, 23–26], which in turn may reduce the risk of recurrent falls in older patients [27].

In our study on caregivers of older blunt trauma patients, negative and positive experiences of caregiving were associated with patient pre-injury functional dependence, and certain patient and caregiver demographics, but not with pre-injury frailty, comorbidity, injury severity, or pattern of injury.

Although our study encompassed a broad range of injury patterns in older patients, the findings are similar to other studies focusing on specific injury patterns. A study on caregivers of patients after traumatic brain injury also found a correlation between caregiver burden and patient disability and executive function, but no correlation with injury severity [28]. Another study on caregivers of older hip fracture patients also showed similar findings to our study, in that caregiver burden increased when the patient had lower function. In addition, caregivers who were already caring for the patient prior to the fracture, experienced higher caregiver burden [29].

While some of the gender-related findings in our study were conflicting, these could be explained by societal norms and expectations of gender roles. Caregivers of male patients were more likely to face financial strain. This could be attributed to the loss of work-related income after injury being more likely for a male patient, as the gender roles in Singapore still reinforce the importance of males as breadwinners [30, 31].

Caregivers of male patients were more likely to lack family support, yet they also reported higher self-esteem. In contrast, male caregivers reported less disturbance to their schedule and health. Taken together, this could mean that female caregivers (e.g., spouses and daughters) of (male) patients were expected to shoulder the burden of caregiving alone, leading to higher self-esteem because they fulfilled a socially expected gender role, and were thus more likely to report lack of family support. A survey of members of the general public conducted in Germany showed more bias against female non-working caregivers, whereas female working caregivers were perceived more favorably [32]. More bias was also reported against male caregivers [32]. While the social dynamics in Germany might be different from Singapore, this suggests that our study findings on gender and caregiving may differ from those in societies with different gender roles and expectations.

Caregivers of functionally dependent patients experienced more financial strain and disturbances in schedule and health. This could be attributed to the physical strain of caring for functionally dependent patients, and the need for higher expenditure (professional caregivers and specialized equipment) to support care at home.

Caregivers of retired patients reported a worse experience with a lack of family support and disturbances in schedule and health. In our cohort, retirees were more likely to have a lower MMSE score and be older than those who were working or homemakers prior to the injury. However, neither age nor MMSE alone were significantly associated with caregiver burden in any of the domains, hence the reasons behind this finding could be more complex.

Not surprisingly, having more people in the household was associated with a less disturbed schedule and health for the caregiver.

Caregivers of patients living in lower socioeconomic/more subsidized housing (compared to those living in private/minimally subsidized housing) were more likely to lack family support. In a study of spinal cord injury patients, lower socioeconomic status caregivers had high overall unmet needs and low psychosocial resources [33]. In addition, other family members could be working longer hours to support the family, and hence may not be able to share the physical burden of caregiving.

One of the limitations of the study was the low recruitment rate and a moderate drop-out rate, possibly due to caregiver stress itself. Despite the low recruitment rate, this was a nationwide multi-centre cohort study of all patients meeting inclusion criteria presenting to public hospitals in Singapore. The demographics of patients presented in Table 1 are similar to the profile of older blunt trauma patients in other studies [5–7, 34], hence we believe that our subjects are representative of our population of interest. However, there are few studies of caregiving in this population of older blunt trauma patients, therefore we could not compare the demographics of our caregivers to those in the literature. Hence our findings may not be generalizable to other populations or settings, although several of our findings are similar to other studies focusing on specific injury patterns) [12, 14].

The strength of the study is that the study tool utilized in this study (21-item modified-Caregiver Reaction Assessment [m-CRA]) is widely used in the literature [1] and has been validated in three of the official languages in our local population [2].

The final limitation of our study was that the survey was primarily to assess the caregiver stress, but we did not design the study to examine possible reasons for relieving or exacerbating stress. More research is indicated on the multidimensional impact of trauma on older patients and their caregivers.

## Conclusions

Negative and positive experiences of caregiving among caregivers of older trauma patients are associated with pre-injury disability and certain patient and caregiver demographics. Good family function, social support, number of caregivers, behavioral intervention, and resilience skills were associated with reduced caregiver burden, while caregivers of more functionally dependent patients, retired patients, and caregivers living in lower socioeconomic housing were associated with higher caregiver burden. These factors should be taken into consideration when planning the post-discharge support for high-risk patients and their caregivers.
